# Effects of Core Executive Function Training on Student Interpreters’ Consecutive Interpreting

**DOI:** 10.3390/bs15111477

**Published:** 2025-10-30

**Authors:** Lan Mao, Qingping Li

**Affiliations:** School of Foreign Languages, Central South University, Changsha 410083, China; qpli@csu.edu.cn

**Keywords:** executive function training, consecutive interpreting, student interpreters

## Abstract

While updating, inhibition, and task switching (TS)—the acknowledged core executive function (EF) components—are implicated in interpreting processing, their distinct roles, particularly for inhibition and TS, are rarely explored within training contexts. This study examined the effects of training different EF components and the durability of such effects on student interpreters’ consecutive interpreting performance. Sixty-four Chinese students were randomly assigned to either an EF training condition (updating, inhibition, or TS) or a control condition. Following five-week training (15 sessions), improvements in the trained EF component and English-to-Chinese interpreting were evaluated, with long-term gains measured after three months. Results showed that while each training condition improved its targeted EF component, only inhibition training produced statistically significant immediate and durable effects in improving interpreting performance. These results are discussed in light of interpreting as an intense and unique bilingual task that substantially relies on domain-general EF components.

## 1. Introduction

Interpreting is an extremely demanding task, requiring the deployment of an array of linguistic and cognitive control mechanisms for the management, coordination, and execution of various seemingly concurrent processes (Hervais-Adelman & Babcock, 2019). Therefore, in addition to the well-established role of language proficiency (e.g., Gile, 2009), domain-general executive function (EF, also cognitive control^1^), an umbrella term that describes a collection of top-down cognitive processes required when a person must concentrate and pay attention (Diamond, 2013), may play multiple important roles in interpreting due to its multi-component feature (Miyake & Friedman, 2012).

Research on EF has begun to establish and verify the significant role(s) of its various components in interpreting by examining their relationship with interpreters’ performance, which observes an overall positive correlation between the two (e.g., Mellinger & Hanson, 2019; Timarová et al., 2014). Nevertheless, EF-related issues are not sufficiently addressed through longitudinal research designs, especially for student interpreters. Compared to professionals, student interpreters are prone to experience cognitive saturation (Cai et al., 2015; Köpke & Signorelli, 2012), making it harder for them to mobilize and coordinate cognitive resources during interpreting (Dong, 2018). Accordingly, the current study focuses on training EF in student interpreters, with particular attention to the three core components generally conceptualized within EF: working memory (WM) updating (hereinafter updating), inhibition, and task switching (TS, also cognitive flexibility). These three components exhibit unity and diversity (Miyake et al., 2000), further raising the question(s) of whether different components contribute uniquely to interpreting processing and/or demonstrate distinct developmental patterns as the training unfolds.

### 1.1. Executive Function in Interpreting: Theoretical Foundations and Empirical Studies

Interpreting taps on complex bilingual cognitive processing and involves managing multiple tasks, such as listening and decoding the input speech, deverbalizing and transcoding from the source language to the target language, planning and reformulating target language delivery, and, in the case of consecutive interpreting (CI), note-taking (Gile, 2009). Interpreters need to make rapid and frequent language conversion under extreme time pressure (Dong & Li, 2020), which places extremely high demands on their cognitive control abilities.

Many influential models of interpreting highlight the significant role of WM, positing that it supports source language comprehension, integration of contextual and semantic information, and the encoding and production of the target language as a larger memory capacity facilitates temporary information storage and processing (Gile, 2009; Mizuno, 2005; Moser, 1978). However, in recent years, research on the cognitive processing in interpreting has shifted from the initial predominant focus on (working) memory to a broader interest in EF (Nour et al., 2020), given the task’s substantial reliance on cognitive control.

For example, updating, namely, the ability to update information held in WM (Miyake et al., 2000), is proposed to be more central in CI than WM capacity. Dong et al. (2018) found that student interpreters’ updating ability (measured by a visuo-spatial 2-back task) in both the pre-test and post-test significantly predicted CI performance, whereas the relationship between WM (measured by an L2 listening span task) and CI performance was weaker. Another study also found that student interpreters’ updating efficacy significantly predict their CI performance (Y. Liu & Dong, 2020). The researchers propose that this may be because updating shares the same attentional control process with the recalling process in CI (Dong et al., 2018). Specifically, after listening to a segment of input, consecutive interpreters must redirect their attention from the end of the segment back to its beginning to retrieve and reconstruct the original message for interpreting. This recall process involves reactivating information that has already passed out of the immediate focus of attention and updating it in real time, engaging domain-general mechanisms similar to those used in updating tasks. In the n-back task, a widely used measure of updating ability, participants are required to recall or make judgments about stimuli presented n trials earlier, mirroring the continuous updating demands faced by interpreters during CI (Dong et al., 2018). This shared processing mechanism is further supported by a recent fNIRS study, which examined the effects of updating training on student interpreters’ CI performance (Öztürk, 2018).

Furthermore, during interpreting, interpreters need to manage two highly activated languages simultaneously to ensure smooth switching between them within a limited time frame. However, the concurrent activation of both languages exposes interpreters to the challenge of cross-linguistic interference (Chmiel et al., 2020). From the perspective of language control, domain-general inhibition serves as a key mechanism for both bilingual language control (Green, 1998) and goal-directed behavior (Zhao et al., 2016). For interpreters, inhibition reduces cross-language interference during source language comprehension and target language production, thereby enabling smooth transitions between languages (Gernsbacher & Shlesinger, 1997; Dong & Li, 2020). Insufficient inhibition may manifest as prolonged lexical retrieval or increased error rates (H. Liu et al., 2017). As an alternative perspective on the role of inhibition in bilingual processing and rooted in the distinguishing features of interpreting processes, i.e., the frequent and swift switching between languages and subtasks, Dong and Li (2020) proposed the Attentional Control Model (ACM) of interpreting, which posits that interpreters’ ability to enhance target language activation and processing can effectively mitigate source language interference.^2^

Interpreting is, by nature, a language conversion task that involves frequent and swift switching between two languages and the underlying information, as well as rapid transitions between different subtasks (e.g., listening, delivery, and note-taking if in CI), all of which may require domain-general TS ability. Therefore, it can be inferred that TS may play an important role in interpreting processing (e.g., Moser, 1978). Nevertheless, the theoretical discussion on TS is rare compared to that of updating and inhibition. Dong and Li (2020) incorporate TS into the ACM, proposing that it supports focused attention and the establishment of language-modality connections, thus enabling linguistic control in interpreting.

Abundant cross-sectional studies employing correlational and/or regression analyses have attested to these discussions, which report that updating is significantly correlate with interpreters’ performance (Dong et al., 2018; Y. Liu & Dong, 2020; Song et al., 2023; Timarová et al., 2014). However, empirical research on inhibition and TS remains limited. For the two independent components of inhibition, response inhibition, and interference inhibition (Diamond, 2013), limited research has only found that interference inhibition is significantly related to interpreting performance (Song et al., 2023; Timarová et al., 2014). In contrast, Xu (2012) reported that interference inhibition did not predict interpreting performance. Taken together, this evidence suggests that the role of inhibition in interpreting requires further investigation. The influence of TS on interpreting also remains inconclusive and awaits further investigation. M. Liu (2014) found a positive correlation between student interpreters’ TS ability and CI performance. Timarová et al. (2014) also suggested TS involvement in simultaneous interpreting (SI) processing, as interpreters with better TS ability keep shorter ear-voice span (EVS; the time lag between the interpreter’s perception of the source text and production of the target language). However, Song et al. (2023) found no significant effect of TS in bidirectional CI, which may be attributed to the specific Chinese–Japanese language pair examined in their study. The shared Chinese character vocabulary between Chinese and Japanese might have highlighted the role of inhibition over that of TS.

However, cross-sectional designs provide restricted developmental information. Some longitudinal studies have examined the predictive power of updating and found it can significantly predict performance in students with less than one year of training (Dong et al., 2018; Y. Liu & Dong, 2020). This provides pedagogical implications for updating training to promote the performance of early-stage student interpreters. Compared to the more extensive research on updating, longitudinal investigations of inhibition and TS remain scarce despite their important roles in language control and processing during interpreting.

Consequently, although a general theoretical consensus exists regarding the link between EF and interpreting, the precise relationship between each EF component and interpreting performance remains unclear. Moreover, current empirical evidence probing this issue relies primarily on correlational analyses and/or cross-sectional studies. In response to the current research, this study implements a comprehensive EF training program targeting the three core EF components in student interpreters. This training approach allows us to clarify the cause-and-effect interplay between distinct EF components and interpreting performance, moving beyond the limitations of previous correlational analyses and bringing the often-overlooked inhibition and TS into the discussion. In addition, the selection of CI as the test domain in the current study is primarily based on practical considerations, namely, that CI is the most basic and commonly practiced interpreting mode in student interpreter training programs (Dong et al., 2018). The participants in this study were receiving CI training during the semester in which the experiment was conducted (detailed in Section 2.1). Although SI is generally regarded as posing higher demands on cognitive control than CI, previous studies have found that CI output is simpler in terms of vocabulary and syntax compared to SI output. This indicates that the cognitive load of CI is not necessarily lower than that of SI (Lv & Liang, 2018).

### 1.2. Executive Function Training in Interpreting

Building on the general recognition of the significant roles of EF in interpreting, EF training is a significant paradigm for clarifying the specific relationship between different EF components and interpreting processing and for understanding cognitive processing in interpreting. An important issue that comes with it is whether EF is trainable. The primary assumption behind this issue is brain plasticity, claiming that EF training could enhance EF development. Numerous studies have reported a positive training effect on EF, evidenced by increased speed and/or accuracy in tasks measuring EF abilities (e.g., Jaeggi et al., 2008; Karbach & Kray, 2009; Thorell et al., 2009; Zhao et al., 2018).

Researchers usually train EF components separately to avoid unnecessary interference. For instance, n-back or running memory training tasks were widely used for updating training (Jaeggi et al., 2008). Inhibition training, especially interference inhibition, which centers on conflict monitoring and target information enhancement (Qi et al., 2021), commonly employs the Stroop task (Wilkinson & Yang, 2012) and the Flanker task (Thorell et al., 2009). TS training mainly adopts a combo task consisting of two subtasks, of which letter classification (vowel/consonant decision) and number classification (odd/even decision) were common ones (Gaál & Czigler, 2018). These training paradigms are designed to minimize individuals’ use of domain-specific strategies and target domain-general mechanisms of EF components (Morrison & Chein, 2011). Hence, many studies have explored whether the training effects could extend beyond strictly controlled laboratory settings to untrained, real-life tasks that heavily rely on EF, such as fluid intelligence (Jaeggi et al., 2008), reading comprehension (Holmes & Gathercole, 2014) and general academic performance (C. Wang, 2019).

Extending these findings in the generalized domains, researchers have considered interpreting as an applied domain of cognition, representing real-life skills for interpreters due to its substantial reliance on EF. For example, Öztürk (2018) found that 14 sessions of updating training improved participants’ CI performance. Zhang and Yu (2018) applied WM capacity and coordination training in SI teaching and affirmed a positive training effect, particularly in the beginning stage. Nevertheless, previous studies rarely report the durability of the training effects, with Öztürk (2018) being a notable exception, showing that the benefits of updating training were maintained three months later. Together, these studies offer evidence that training on students’ cognitive ability could transfer effectively to complex applied domains such as interpreting.

In comparison to the relatively more frequently examined role of updating training in interpreting, the training effects of inhibition and TS are less clear, even though both are recognized as important in bilingual processing. A higher inhibition ability facilitates faster suppression of the current language and activation of the target language during language switching in bilinguals with comparable proficiency, thus incurring a smaller language switching cost (Green, 1998). Its training has shown transfer to the language domain, as studies have reported that inhibition training significantly reduced the language switching cost in the picture naming task (Chang et al., 2018; H. Liu et al., 2016). For TS, it holds a significant role in complex L2 processing tasks, such as listening (Yang et al., 2022) and reading (Relyea et al., 2023), which require attention shifting across many different tasks (e.g., phonological processing, constructing semantic representations) and multiple aspects of languages simultaneously (e.g., phonological, lexical, grammatical patterns). Therefore, improved domain-general TS ability might facilitate flexible language switching. Collectively, this evidence demonstrates that domain-general core EF training can enhance bilingual performance across language comprehension and production tasks. This interdependence provides a solid foundation for examining EF training in interpreting.

Interpreting, after all, differs from general bilingual processing in its frequent and rapid switching between two languages, which necessitates the alternating suppression and activation of each language. In this context, the overlap between domain-general EF mechanisms and language control becomes even more pronounced. This process might tax more on inhibition and shifting, raising the need to further clarify their roles in interpreting. In addition, some studies have reported delayed effects of EF training, referring to positive outcomes observed in the delayed post-test rather than immediately after training (C. Wang, 2019; Zhao et al., 2018), thereby highlighting the need for follow-up measurements. Consequently, the current study integrates the three core components under the same training protocol and tracks whether the training effects are sustained and stable, minimizing unnecessary variable interference and providing pedagogical implications for student interpreter training.

### 1.3. The Current Study

In response to the literature review (major findings are summarized in Table 1), the current study was designed to train the three core EF components, i.e., updating, inhibition, and TS, in student interpreters. Training treatments would run in three groups separately, each receiving training on one component. This study first examined the effect of training on the performance of the trained EF component, based on which the training effects on students’ CI performance were then explored. The durability of these training effects (if any) was also investigated. The specific research questions (RQs) and hypotheses (H) are:

RQ1: Is training on individual EF components (updating, inhibition, and TS) effective in improving performance in corresponding untrained cognitive tasks measuring the same construct? Are these training effects, if any, durable?

**H1:**  *Training in updating, inhibition, and TS will significantly improve participants’ performance on corresponding EF tasks, and these gains will be sustained at follow-up*.

RQ2: Is training on individual EF components differentially effective in improving student interpreters’ CI performance? Are these training effects, if any, durable?

**H2:**  *Training in updating, inhibition, and TS will have positive, immediate, and sustained effects on students’ CI performance, with relative effects for the four groups in the order: Updating = Inhibition = TS > Control*.

## 2. Methods

### 2.1. Participants and Background

Seventy Chinese undergraduates majoring in interpreting participated in this study. English is their foreign language (L2). Interpreting in this study hereinafter refers to the consecutive interpreting of L2–L1 (English–Chinese). Before the study began, the participants had received one semester (16 weeks) of interpreting training, consisting of one 90 min interpreting class per week (including a 10 min break). According to the school’s course schedule, they were also required to take a second semester (16 weeks) of interpreting training during the period in which the study took place, following the same arrangement of one 90 min weekly class (including a 10 min break). At the beginning of the study, the participants had already completed the first week of their second-semester interpreting course. All participants came from two intact classes in the same academic year and received interpreting instruction from the same teacher throughout the study.

Interviews with the teacher indicated that, during the previous semester, the interpreting course primarily focused on liaison interpreting, organized by specific topics (e.g., ceremonial speeches, international exchanges). Regular class time was predominantly devoted to lectures on interpreting strategies and related practice. Classroom observation during the experimental semester (i.e., the second training semester) revealed that the interpreting exercises and lectures mainly employed CI, with occasional sight translation. In such cases, after the participants practiced interpreting a CI segment, the teacher displayed the transcript of the reference translation on the screen and asked students to perform back-translation exercises.

All participants were randomly assigned to a training or a control group. Six students either quit or did not finish all training sessions (one from the Inhibition group and five from the TS group), resulting in the final grouping as follows: Updating group (*n* = 17), Inhibition group (*n* = 16), TS group (*n* = 13), and Control group (*n* = 18). The four groups were matched for age, linguistic proficiency, handedness, and cognitive measure (Table 2). All participants signed a written informed consent and received monetary compensation. The study was conducted in accordance with the ethical standards of the participating university.

### 2.2. Study Design

This study adopted a pre-/post-/delayed post-test design to investigate the training effects of different EF components in building EF abilities and promoting interpreting performance, as well as the possible durability of these effects. Participants were required to take an assessment battery before EF training (pre-test, T1), immediately after training (post-test, T2), and three months after the post-test (delayed post-test, T3). During the three-month interval between T2 and T3, which included the winter vacation (about 45 days), the participants did not receive any planned interpreting or EF training.

Each assessment battery includes three cognitive ability tasks and one interpreting task. Participants completed all tasks on two consecutive days in the following order. On day one, they performed cognitive ability tasks in a quiet language lab, which took about 35 min together. On day two, they took the interpreting task, which lasted 10 min, similar to the practices in their interpreting courses.

### 2.3. Training Protocol

This study administered adaptive running memory tasks (Updating group), adaptive Stroop and Flanker tasks (Inhibition group), and an adaptive task-cueing switching task (TS group) to three training groups. The adaptive design was adopted for all training tasks as this design allows task difficulty to adapt to the participant’s actual performance, thus better catering to individual differences, which is more effective for training (Klingberg et al., 2005). All tasks were programmed using E-Prime 2.0 software. The training was conducted outside regular class hours, and the participants needed to finish 15 computerized program sessions (three sessions per week for five weeks) in a quiet language lab, each lasting 20–25 min. This schedule was informed by prior cognitive training studies and was considered manageable given participants’ academic routines. The Control group followed a routine course schedule without any planned EF training.

#### 2.3.1. Adaptive Running Memory Tasks

The Updating group received two adaptive running memory training tasks: letter and animal running memory tasks patterned after Zhao et al. (2013). The two tasks differed only in stimuli materials (letter or animal). For example, in the letter running task, a fixation “+” was presented for 800 milliseconds (ms) in the center of the screen to signal task onset. Then, some letters appeared consecutively, with the total number varying 5, 7, 9 or 11 randomly between trials. Participants needed to remember the last three letters in the presented order, type them in the answer box, and confirm their answer by clicking “Confirm” to start the subsequent trial. Since participants could not predict the total letter number in the current trial, they needed to constantly hold and update the information and keep remembering the last three letters whenever a new letter appeared. Correct answers scored one point, and wrong answers were not scored.

Participants were required to complete six blocks of five trials for each training session, with breaks between blocks if needed. Each letter’s presentation time (PT) was 1750 ms at the beginning of training. Adaptive design was achieved as task difficulty varies with the participant’s performance. Within each block, if participants scored three or more out of all five trials, the PT of letters in the subsequent block would decrease by 100 ms (more difficult). Otherwise, it would increase by 100 ms (less difficult). The baseline difficulty and adaptive mechanisms for these tasks followed the procedures established in Zhao et al. (2013), where healthy young adults completed comparable adaptive updating training.

#### 2.3.2. Adaptive Stroop and Flanker Tasks

The Inhibition group took adaptive Stroop and Flanker tasks modeled after Thorell et al. (2009). Stroop stimuli were created from 4 Chinese color characters (红 RED, 绿 GREEN, 黄 YELLOW, 蓝 BLUE) presented individually in one of the four colors (red, green, yellow, blue), forming two types of trials, congruent (the ink color of the character is consistent with its meaning, such as 蓝 BLUE in blue ink) and incongruent (the ink color of the character is different with its meaning, such as 蓝 BLUE in green ink). Each trial started with a fixation (500 ms) and a blank screen (1000 ms). Then, the target stimulus (color character) was presented. Participants were required to respond to the print color of the word by pressing D for red, F for green, J for blue, and K for yellow. The subsequent trial started immediately after the participant’s response. Task difficulty was manipulated by adjusting the time of response window, which in turn would affect individual performance and enable adaptive design. For each training session, participants were required to complete three blocks of 80 trials each, with congruent and incongruent trials equally distributed (50% each). If the accuracy of the current block was less than 75%, the response window in the next block would increase by 100 ms; if it was above 90%, it would decrease by 100 ms. The response window would stay the same if the percentage of correct responses ranged from 75 to 90%. Initial training started with a 2000 ms response window. The baseline difficulty and adaptive structure were set based on prior studies using university student samples (e.g., Zhao & Jia, 2019), allowing comparability across training groups in task demand and adaptive progression.

In the Flanker task, five arrows pointing right or left were presented in a row. The arrow in the middle was the target, and the other four were distractors. Participants should identify the direction of the target arrow by pressing J for the right and F for the left. Task details were the same as those in the Stroop task.

#### 2.3.3. Adaptive Task-Cueing Switching Task

The TS training task was adapted from Kray and Fehér (2017) and Gaál and Czigler (2018). The baseline difficulty and adaptive adjustments followed the procedures reported in these studies, and a univalent design was employed to reduce the inhibition component in the process (Dong & Liu, 2016). Target stimuli were digit-letter combinations, e.g., G6. The cue was the ink color of the target stimulus (green or black). The black color signaled that the participant needed to perform Task A (odd/even judgment) by pressing F for an odd or J for an even number. The green color indicated that the participant needed to complete Task B (vowel/consonant judgment) by pressing B for a vowel or N for a consonant. The formal training consisted of 5 blocks of 73 trials. The first one was fixed as Task A (excluded from analysis), and the remaining 72 trials consisted of a pseudorandom mix of Task A and Task B with a 50% switch probability.

This task achieved adaptive design by adjusting the stimulus PT. Each trial began with a fixation “+” (500 ms), followed by the target stimulus with a 2000 ms initial PT. If the accuracy in the present block was higher than or equal to 85%, the stimulus PT in the next block was reduced by 100 ms; if lower than 60%, the PT would increase by 100 ms. Stimulus PT remains unchanged if the accuracy rate is between 60–85%.

### 2.4. Assessment Battery

#### 2.4.1. EF Ability Tasks

This study employed three EF ability tasks to collect participants’ EF baseline and training effects data. In line with previous studies (Gaál & Czigler, 2018; Öztürk, 2018; Zhao et al., 2018), for each EF component, we used assessment tasks with the same structures as described for the training tasks but involved different target stimuli and/or trial conditions. Specifically, we used a 2-back task to measure the changes in updating ability before and after training, a Stroop task with neutral trials to test inhibition, and a TS task consisting of number magnitude judgment and letter case judgment to measure TS ability. Detailed task descriptions and dependent measures of these tasks are presented in the Supplementary Information.

#### 2.4.2. CI Tasks and Scoring

The interpreting task was conducted in a classroom with recording equipment. We used three materials at different test slots to avoid the practice effect. Table 3 presents the features of the three interpreting materials used at the three test points (See the Supplementary Information for full transcripts of the CI speeches).

**Figure 1:**
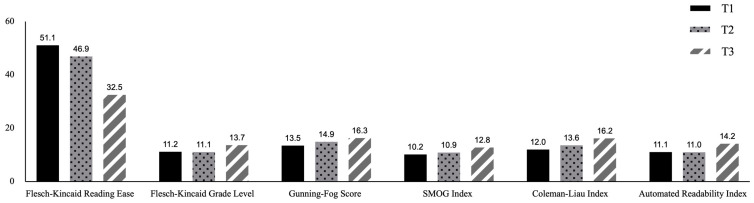
Readability indices of the materials.

The three materials were of an equivalent level of difficulty based on the following evidence. (1) All materials were selected from the original materials of CI Level Three China Accreditation Test of Translators and Interpreters (http://www.catticenter.com/), which were designed to test general interpreting competence and the topics were commonly covered by interpreter training programs, such as China–US cooperation, posing no requirements on terminological preparation. (2) Three experienced interpreting instructors agreed that the three materials exhibited no notable difference in general interpreting difficulty (language- and topic-wise). (3) The materials showed comparable results in text readability indices (https://www.webfx.com/tools/read-able/) (accessed on 29 December 2024) (Figure 1), and overall speech features (Table 3).

This study employed a relatively refined approach to assess participants’ CI performance, namely, by analyzing the presence and performance of idea units (Christoffels et al., 2003; M. Liu, 2014). This method was chosen because it could capture the amount of information that a participant can render within a limited time more accurately.^3^ Two scorers, both experienced interpreting instructors and practitioners, divided each speech into several idea units to separate meaningful ideas. They then rated each idea unit from 0 to 4. A score of 0 means that the idea unit was completely missing from interpreting; a score of 1 means that the idea unit appeared, but most of the information was missing or incorrect, and so on, up to a score of 4, which indicates that the idea unit was interpreted in its entirety correctly. The score was then summed, standardized, and presented in the form of 100 points.

The two scorers first rated 20 recordings independently, and when a discrepancy in the final score was over 5, they would discuss it until the discrepancy was solved. Then, they proceeded to rate the remaining recordings independently. Inter-rater reliability was excellent, as indicated by both a Pearson’s correlation (*r* = 0.908) and an intraclass correlation coefficient (ICC) = 0.967, 95% CI [0.923, 0.986]).

## 3. Results

### 3.1. Training Effects on EF Ability

To monitor whether participants were engaged and exerted cognitive efforts in the training tasks, this study tracked their progress across 15 training sessions, as a supplementary measure of the training effects on EF abilities. This was assessed by examining changes in task difficulty, which were dynamically adjusted based on individual performance, either through adjustments to the presentation time of the stimulus or to the time allowed for response (detailed in Section 2.3).

Training data were analyzed using repeated measures analysis of variance (ANOVA), with Session (1–15) as a within-participant factor. Data were processed using SPSS 29.0. All post hoc comparisons were based on estimated margin means with a Bonferroni correction at *p* < 0.05 throughout, and partial eta-squared (η_p_^2^) was used as an estimate of effect size. The same analytic approach and correction procedures were applied consistently across all reported analyses.

**Figure 2:**
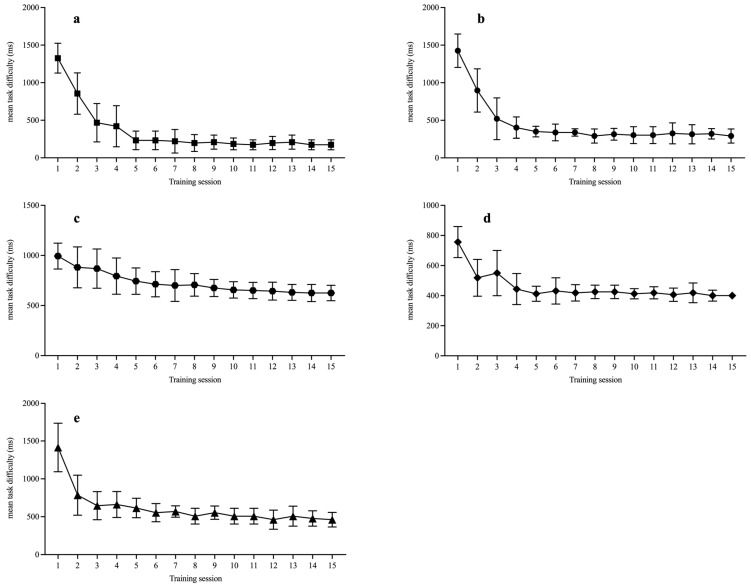
Mean task difficulty of the adaptive training tasks: (**a**) Letter running memory task; (**b**) Animal running memory task; (**c**) Stroop task; (**d**) Flanker task; (**e**) Task-cueing switching task.

Figure 2 illustrates the performance of the three training groups during 15 training sessions. In all task types, the mean task difficulty decreased over time, indicating progress during training, a finding supported by the ANOVA results (Table 4), which revealed significant main effects of training session for each task type. This suggests that participants fully understood their respective task(s) and dedicated substantial cognitive resources to their completion.

For each trained EF component, pre- and post-training performance and potential durable training effects were analyzed based on the dependent measure from the specific assessment task, using Group (Updating, Inhibition, TS, Control) and Time (T1, T2, T3) repeated measures ANOVAs. Descriptive statistics are summarized in Table 5. Analyses of accuracy data across all EF tasks revealed no significant within- or between-group differences, suggesting that the observed reaction time effects were not confounded by speed–accuracy trade-offs. Therefore, reaction time (RT) was used as the primary indicator of the core EF components assessed.

As can be seen in Table 5, the performance of the three training groups on the corresponding untrained cognitive task measuring the same construct was improved. Notably, the training also seemed to produce cross-component transfer effects. For example, the RT of the Inhibition and TS groups in the 2-back task decreased significantly; the Stroop effect of the TS group decreased significantly; and the values of the mixing cost in the TS task also decreased significantly in the Updating and Inhibition groups. However, since one of the RQs in this study concerns whether training in a specific EF component can enhance performance in the corresponding EF ability, the present study did not address potential cross-component transfer effects of training. This issue, which represents an important topic in cognitive training research, will be discussed in a separate article. Instead, the following section focuses on whether the targeted EF component itself improved as a result of training (RQ1), with comprehensive practical effects and effect sizes for all three EF ability tasks reported in the Supplementary Information.

**Figure 3:**
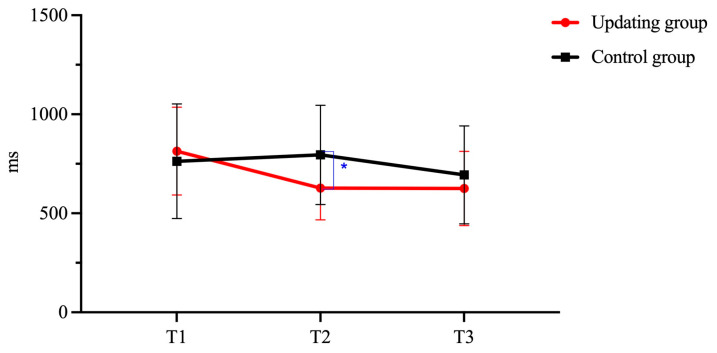
Changes in 2-back task performance before and after training. Note. Error bars represent SD. * indicates *p* < 0.05. Sample sizes (Updating: *n* =17; Inhibition: *n* = 16; TS: *n* = 13; Control: *n* = 18) apply to all figures herein.

Figure 3 presents the changes in 2-back RT for the Updating group and the Control group before and after training. There was a significant Group and Time interaction in the 2-back task (*F* = 6.43, *p* < 0.001, η_p_^2^ = 0.24). Pairwise comparison analyses revealed that the Updating group and the Control group showed no statistical difference at T1 (*p* = 1.000), suggesting the two started out equally. After 15 sessions of training, the Updating group significantly outperformed the Control group at T2 (*p* = 0.042), suggesting an immediate improvement in updating ability. However, this advantage disappeared at T3 (*p* = 1.000). Moreover, the within-group comparisons showed that the Updating group showed a significant within-group development with the pattern T2 > T1 (*p* < 0.001), T3 > T1 (*p* < 0.001), T3 = T2 (*p* = 1.000). The Control group demonstrated no immediate improvement at T2 when compared to T1 (*p* = 1.000), but performed significantly better at T3 when compared to T2 (*p* < 0.001).

**Figure 4:**
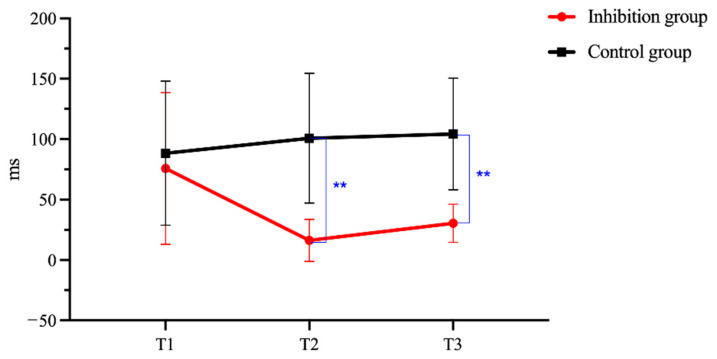
Changes in Stroop task performance before and after training. Note. ** indicates *p* < 0.01.

There was a significant interaction between Group and Time in the Stroop task (*F* = 4.31, *p* < 0.001, η_p_^2^ = 0.18), supporting the positive training effects in improving participants’ inhibition ability. Figure 4 presents the changes in Stroop effect for the Inhibition group and the Control group before and after training. Pairwise comparison analyses demonstrated that the Inhibition group and the Control group had no statistical difference at T1 (*p* = 1.000). However, the Inhibition group was significantly better than the Control group at both T2 (*p* < 0.001) and T3 (*p* < 0.001), indicating that inhibition training produced an immediate development in inhibition ability, and the training effect was durable for three months. Moreover, the within-group comparisons showed different developmental patterns for the two. The Inhibition group boasted significant within-group development with the pattern T2 > T1 (*p* = 0.001), T3 > T1 (*p* = 0.015), T3 = T2 (*p* = 0.817). There was no statistically significant within-group change for the Control group (T3 = T2 = T1, *p*s > 0.05).

**Figure 5:**
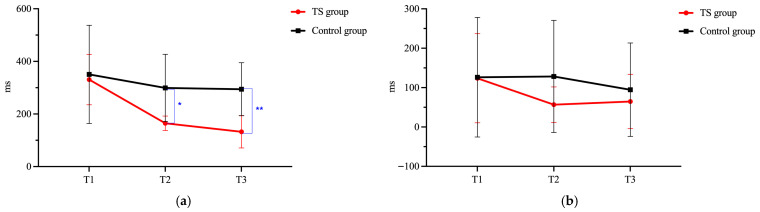
Changes in TS task performance before and after training: (**a**) Mixing cost; (**b**) Switching cost. Note. ** indicates *p* < 0.01; * indicates *p* < 0.05.

Between the two dependent variables of the TS task, only a significant interaction between Group and Time has been found for the mixing cost (*F* = 3.02, *p* = 0.009, η_p_^2^ = 0.13). Pairwise comparison analyses showed that the TS and the Control group generated equal mixing cost at T1 (*p* = 1.000). TS training produced immediate and durable training effects in reducing mixing cost, as evidenced by the significant improvements for the TS group at both T2 (*p* = 0.003) and T3 (*p* < 0.001) compared to the Control group. Moreover, the within-group comparisons showed that the TS group had significant within-group development with the pattern T2 > T1 (*p* < 0.001), T3 > T1 (*p* < 0.001), T3 = T2 (*p* = 0.632). Yet, the Control group had no statistically significant development despite the continuous decline in the mixing cost value (T3 = T2 = T1, *p*s > 0.05). Figure 5a presents the changes in mixing cost for the TS group and the Control group before and after training. There was no significant interaction between Group and Time for the switching cost (*F* = 0.38, *p* = 0.888, η_p_^2^ = 0.02). Post hoc comparisons yielded no meaningful contrasts. The TS group did not differ significantly from the Control group at any of the three time points (*p*s > 0.05). And the two groups showed no significant within-group variation over time (*p*s > 0.05). Figure 5b presents the changes in switching cost for the TS group and the Control group before and after training.

### 3.2. Training Effects on CI

**Figure 6:**
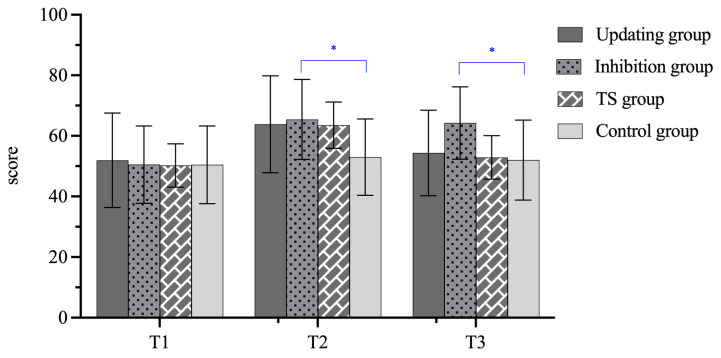
Changes in CI performance before and after training. Note. * indicates *p* < 0.05.

Descriptive statistics for CI scores are also summarized in Table 5, while Figure 6 illustrates the changes in CI performance scores across all groups before and after training. Table 6 summarizes the practical effects and effect sizes for interpreting performance. The repeated-measures ANOVA results revealed a significant Group × Time interaction effect (*F* = 22.38, *p* < 0.001, η_p_^2^ = 0.528). Pairwise comparison analyses revealed that the simple main effects for Group at T1 were not significant (*p*s = 1.000). By contrast, the Inhibition group performed significantly better than the Control group at T2 (*p* = 0.044), while the other two training groups showed higher scores than the Control group, though differences did not reach significance (Updating group, *p* = 0.098; TS group, *p* = 0.178). These findings indicate that the four groups started equally and that all training groups were better than the Control group at T2, but only inhibition training reached statistical significance. The effectiveness of inhibition training continued to be visible at T3 (*p* = 0.031), suggesting a durable improvement for inhibition training. The Updating and TS training groups’ advantages over the Control group were non-significant at T3 (*p*s = 1.000). The three types of EF training tasks were equally efficient at T2 and T3 (Updating group = Inhibition group = TS group, *p*s > 0.05).

Moreover, the within-group comparisons showed different developmental patterns for each group. The Updating and TS groups showed similar performance trajectories. Both demonstrated a significant improvement in CI performance at T2 compared to T1 (T2 > T1, *p*s < 0.001). However, their scores declined at T3, falling significantly below those at T2 (*p*s < 0.001) but not differing significantly from those at T1 (*p*s > 0.05). By contrast, the Inhibition group showed continuous within-group developments (T3 > T2, T3 > T1, *p*s < 0.001). The Control group showed no natural gains over time in interpreting (T3 = T2 = T1, *p*s > 0.05).

## 4. Discussion

In light of the important role of each core EF component in interpreting processing, this study conducted EF training on student interpreters to examine whether the training on individual EF component improved the corresponding EF ability and to explore further the effects of training on student interpreters’ interpreting performance. The findings provide a confirmative answer to RQ1: training on individual EF components effectively improved the corresponding EF abilities. However, only the effects of inhibition and TS training remained durable in the delayed post-test three months later, thus partially supporting Hypothesis 1. Regarding RQ2, the results did not support our second hypothesis. Although all three training groups outperformed the Control group in the immediate post-test, only the effects of inhibition training reached statistical significance and remained stable over the three-month delay. Pairwise comparisons among the training groups did not reveal significant differences; however, distinct within-group developmental patterns emerged, with only the Inhibition group maintaining its improvements over time. The results are expected to present empirical evidence for the role of different EF components in interpreting and shedding light on related developmental issues.

### 4.1. Improvements in EF Abilities

The three types of training tasks, each targeting one of the core EF components, all adopted adaptive designs. These designs were achieved by manipulating task difficulty, either by adjusting the stimulus presentation time or the response window based on participants’ performance. Previous studies have shown that adaptive training designs are more effective in accommodating individual differences and maximizing training outcomes (Klingberg et al., 2005). In the present study, the average task difficulty for the three training groups decreased significantly as the training progressed, which is consistent with findings from previous studies using similar training tasks (e.g., Chen et al., 2018; Zhao & Jia, 2019; Zhao et al., 2020). Although the observed progress during training suggests that participants exerted substantial cognitive effort, it remains necessary to examine their performance on untrained tasks to determine whether the training effectively enhanced the targeted EF components.

After five weeks of training (15 sessions), all three training groups demonstrated immediate improvements in EF tasks that were structurally similar to their respective training task(s). The positive training effects are consistent with earlier behavioral results with EF training in healthy young adults (e.g., Chang et al., 2018; Gaál & Czigler, 2018; Öztürk, 2018). One likely explanation for these improvements is that participants developed task-specific strategies during training (Jaeggi et al., 2008; Zhao et al., 2020), which were transferred to the assessment tasks.

Specifically, the running memory task requires participants to continuously encode new stimuli while updating previously memorized items in the sequence in which they appear (Zhao et al., 2016). Training on this task may first enhance stimulus recognition, then improve attentional focus on the current target stimulus, thereby strengthening the updating process (Zhao et al., 2013). The 2-back task used for assessment also emphasizes the updating and manipulation of information. It involves a range of operations, including the active maintenance of items and their serial order in WM, repeated encoding of the current item, comparison with items n trials back, and continuous updating of the list (Öztürk, 2018).

As for inhibition, apart from the inclusion of a neutral condition, designed to prevent participants from becoming aware of the stimulus presentation rules and to diversify the task conditions (Dong & Chen, 2020), the measurement and the training tasks are structurally consistent. During training, participants were trained to ignore interfering visual information, inhibit incorrect prepotent responses associated with that interference, and more effectively focus on task-relevant target information (Zhao & Jia, 2019). Taking the Stroop task as an example, the conflict arises from the competition between the stimulus’s color (target) and its word meaning (interference). Successful task performance requires participants to name the color while ignoring the word’s semantic content. This process engages top-down cognitive control mechanisms, including the classification of stimulus elements into target and interference components. Through active regulation, participants learn to selectively enhance attention to the target color information, suppress the irrelevant word meaning, and perform the task more efficiently (Zhao et al., 2016).

For TS ability, the training task and measurement task used in this study differ only in subtasks but share the same task structure. Considering both mixing and switching costs are valid indicators of TS ability, the effectiveness of TS training remains supported despite the absence of switching cost improvement. These results suggest that the two were different indices within the TS construct. Mixing cost reflects overall conflict monitoring abilities (Dong & Liu, 2016; Dong & Chen, 2020), proactive control (Braver et al., 2003), and the ability to maintain and monitor multiple task sets (Zhao et al., 2020), while the switching cost reflects the cognitive reconstruction required at the moment of switching and is thought to partly measure interference from the previously active task set (Salminen et al., 2012). Therefore, the divergent findings between the two indicators are plausible. Furthermore, the training task was designed with univalent response buttons to reduce the engagement of the inhibition component, thereby minimizing the need to suppress one task in favor of another. As a result, the absence of improvement in switching cost is understandable, since the training did not actively promote inhibitory control over competing task sets (Salminen et al., 2012).

Another possible reason for the positive training effect is that the training enhanced general attentional control efficiency (Wilkinson & Yang, 2012; Chein & Morrison, 2010). For instance, in the updating training, participants learned to focus attention on the current target, a foundational skill for effective subsequent updating. In the inhibition training, they practiced selecting and attending to task-relevant information while ignoring distracting or irrelevant stimuli. In the TS training, sustained attention to task rules was essential to ensure accurate and rapid transitions between subtasks. Such enhancement in general attentional control efficiency may also lead to cross-component transfer, meaning that training not only improves the construct directly targeted by the task, but may also extend its benefits to related cognitive constructs (e.g., updating training enhancing inhibition) or to broader behaviors associated with the trained construct (e.g., the interpreting task examined in this study). As indicated by the descriptive data in the present study, the observed training effects may not have been confined to a single targeted executive function within each group. However, stronger evidence for potential cross-component transfer effects would require direct empirical examination. Future research should therefore investigate these transfer effects among executive functions more systematically to clarify the functional interdependencies and overlapping processing mechanisms underlying EF components.

At the same time, analysis of the training tasks across different EF components reveals that each type of training has its own specific focus and characteristics. This is consistent with the finding that the three groups showed different developmental trajectories with only the gains from inhibition and TS training maintained for three months. This finding is a valuable contribution to the current limited research on the maintenance effect of EF training (Gaál & Czigler, 2018; Rebok et al., 2014). Together, these findings provide a solid foundation for subsequent investigations into the effects of EF training on interpreting, as will be discussed in the following section.

### 4.2. EF Training and Interpreting Performance

#### 4.2.1. EF Training Generated Immediate Improvements in CI

Building on the practical effects of training on corresponding EF abilities, we are more interested in whether domain-general EF training could improve interpreting performance, an applied domain of cognitive processing. The three types of EF training tasks all produced immediate beneficial effects compared to the Control, suggesting that each EF component training has the potential to facilitate immediate interpreting improvements. Specifically, only inhibition training produced a statistically significant effect, while updating and TS training effects did not reach statistical significance. These positive training results in interpreting may be because the three types of training tasks enhanced general attentional control efficiency, as also reflected in the immediate improvement in the trained EF abilities. This supports the view that language systems cannot be entirely dissociated from these domain-general core cognitive functions (Hervais-Adelman & Babcock, 2019). Given that the execution of interpreting concerns intense language and processing control, it inevitably taps into these domain-general EF components, implying shared cognitive mechanisms underlying the domain-general EF training tasks and interpreting.

Previous studies propose that updating engages processing mechanisms that overlap with those required in CI tasks (Dong et al., 2018; Y. Liu & Dong, 2020). Specifically, the constant updating of information held in WM recruits processes such as encoding, covert maintenance, updating, and recall (Chein & Morrison, 2010). Likewise, successful CI requires the recalling and updating of previously processed information (Dong et al., 2018). However, the non-significance of updating training contrasts with previous training studies in interpreting, which found that WM or updating training improves interpreting quality due to enhanced memory capacity (Öztürk, 2018; Zhang & Yu, 2018). The inconsistency may stem from their training task’s potential bias toward the training group. For instance, Zhang and Yu (2018) employed a memory training task that also engaged language processing, i.e., listening comprehension and key information identification, mirroring the demands of the interpreting task. Alternatively, their assessment task may have emphasized updating. Öztürk (2018), for example, used a memory-only interpreting task, requiring participants to perform CI without taking notes, thereby prioritizing updating’s role. Unlike these two studies, the current study disentangled updating and language processing by using a training task with minimal linguistic elements, focusing only on the role of updating as a domain-general EF component. In addition, the current study used a standard CI assessment task that allows note-taking, as in most interpreting studies (e.g., Cai et al., 2015; Dong et al., 2018), which could have relieved some of the memory pressure.

More importantly, the current study contributes findings beyond the much-studied updating, demonstrating the significant role of inhibition in interpreting with training evidence. Inhibition training improved students’ interpreting performance, consistent with previous correlational and regression research (Timarová et al., 2014; Song et al., 2023). The training tasks used in this study concentrated on target accessing and significantly enhanced participants’ inhibition efficiency, referring specifically to the accuracy of inhibiting irrelevant information and accessing target information, as reflected by the improved performance in the Stroop task after training. A higher inhibition efficiency can help students avoid interference from the source language during target language production (Gernsbacher & Shlesinger, 1997) more efficiently and activate equivalence words faster and more accurately. This is particularly relevant for unbalanced bilinguals performing an L2-L1 interpreting task (as in this study), as this direction of interpreting requires more potent inhibition of L1 when processing the source language (L2) (Green, 1998).

Building upon existing evidence supporting the role of inhibition in bilingual processing, the advantage observed in the inhibition training group suggests that CI places substantial demands on inhibition, particularly when considered in light of the specific cognitive and operational demands of CI. This demand may account for the significant improvement observed in that group. CI typically involves two phases: the comprehension phase and the reformulation phase (Gile, 2009). A distinctive feature of input comprehension in CI is that, while interpreters are actively listening to the input, target language representations may simultaneously be activated (J. Lin et al., 2015), and note-taking occurs concurrently. During this phase, interpreters must identify appropriate equivalents—whether lexical items in the co-activated target language or symbolic representations—for use in their notes. Those with stronger inhibition ability are better able to quickly select and attend to task-relevant information from among multiple simultaneously activated potential equivalents. Conversely, insufficient inhibition may lead to longer target access times, increased error rates (H. Liu et al., 2017), or delays in selecting suitable target symbols during note-taking. These difficulties could negatively affect comprehension, impair efficient note-taking, and ultimately result in inappropriate pauses during output (manuscript in preparation), reduced information quality, or even failure to complete the language conversion process (de Bot, 2000). In the reformulation stage, although cognitive load from simultaneous task processing is reduced (Gile, 2009), interpreters must retrieve and reformulate the target language based on previously constructed source language representations (Song et al., 2023). Thus, inhibition training could influence performance and contribute to the final CI outcome.

Turning to the TS component. A CI task involves switching between different subtasks and between different languages, leading to the common assumption that TS plays a significant role in CI. In this study, TS training led to improved CI performance in the immediate post-test, yet it failed to reach statistical significance when compared to the Control group. This finding echoes a recent study showing the non-significant predictive role of TS in student interpreters’ interpreting scores (Song et al., 2023). Similarly, Timarová et al. (2014) found that interpreters’ TS ability correlated only with their EVS, but not with any of the other seven performance measures, suggesting that TS may primarily work in background processes with minimal impact on final output. Although the TS training task in the current study highlighted cognitive flexibility to rapidly shift attention between tasks (odd/even or vowel/consonant decision task), the result contrasts with M. Liu (2014)’s finding of a significant correlation between TS ability and CI performance in student interpreters. Taken together, the current non-significant advantage of TS training supports the claim that TS likely contributes to back-end processing (Timarová et al., 2014) when interpreters shift between tasks or languages. This is consistent with evidence that the neural mechanisms of language switching and domain-general cognitive TS exhibit a degree of similarity (Abutalebi & Green, 2007), especially in the concurrent maintenance of multiple task rules and information active for upcoming shifting (Zinke et al., 2012). Nonetheless, extending this effect to significantly influence final interpreting output appears challenging. Interpreters are typically familiar with the target task or language they are switching to, as the fixed language-modality connections between input and output in CI are already stored as a task schema through training and experience (Dong & Li, 2020), thereby reducing the cognitive demands of maintaining target task rules during switching.

#### 4.2.2. The Sustained Effectiveness of Inhibition Training

Notably, EF training generated different developmental patterns regarding different components, among which inhibition training showed the most significant potential. Its training effects were sustained three months after the training ceased. This finding was noteworthy because the current study was the first to present training evidence for the impact of inhibition on interpreting and the durability of these effects. Lacking comparable longitudinal studies of inhibition training in interpreting research, we draw on related fields to obtain information regarding its strong effectiveness and development.

Inhibition training has also been shown to produce significant improvements in language processing tasks, particularly among unbalanced bilinguals (Chang et al., 2018; H. Liu et al., 2016), aligning with its established role in bilingual processing (e.g., Green, 1998). However, this is indirect evidence. Given the unique feature of CI, namely, the frequent and regular language switching (Dong & Li, 2020), further longitudinal studies are warranted. Instead of examining each component separately, the current study took the initiative to investigate all three EF components within the same framework in interpreting research. The different roles of each EF component in CI processing would be elaborated in relation to the two-phase feature of CI.

During the comprehension phase, unbalanced bilinguals (such as student interpreters) would allocate significant cognitive resources to comprehend rather than remember source information (J. Lin et al., 2015). Memorizing incomprehensible information does not aid subsequent interpreting but instead risks exceeding cognitive capacity,^4^ causing a spillover effect (Plevoets & Defrancq, 2020). In this phase, inhibition functions primarily in reducing interference and facilitating goal-directed behavior, namely, efficient source language decoding and note-taking. This is particularly crucial in L2-L1 interpreting (as in this study), where suppressing the potent L1 is essential for efficient and accurate L2 decoding (see also the above section). True language switching (target language production) has not yet started at this phase, so the demand for TS is not strong. During the reformulation phase, when interpreting into L1, although the processing of the target language (L1) is unlikely to be interfered by L2, as the non-selective activation of L2 is unlikely, inhibition may mainly work through rapid access and retrieval of the target information, as well as the monitoring of output. These abilities are more strongly associated with inhibition (see Zhao et al., 2016, for a review). The Stroop and Flanker tasks used in training concentrate on accessing target stimuli, thereby improving participants’ ability to inhibit irrelevant information and retrieve appropriate translation equivalence.

In summary, the present study provides direct training evidence supporting the indispensable role of central EF in the language processing system (Mizuno, 2005). However, the three core EF components of interest exhibited different effect strengths and developmental patterns, further echoing the notion of the unity and diversity of executive functions (Miyake & Friedman, 2012).

These findings should be interpreted with caution, as this study has several limitations. The primary limitation is the relatively small sample size. Small participant cohorts are a common critique in empirical interpreting research (Dong & Chen, 2020), as they may limit the generalizability of the findings. The sensitivity analysis indicated that the current design was sufficiently powered to detect small-to-medium interaction effects (η_p_^2^ ≈ 0.03–0.06), although smaller effects may not have reached statistical significance. Nevertheless, this limitation should be understood within the context of student interpreter training programs, which are typically highly selective and maintain small annual intakes to ensure intensive, high-quality training. For instance, in China, annual enrollment in Bachelor of Translation and Interpreting (BTI) programs generally ranges from 20 to 50 students per grade per institution (B. Wang & Mu, 2009). This limited enrollment base reduces the pool of potential participants, making it difficult to recruit large samples from a single institution while also controlling for confounding variables such as differences in teaching methodologies and curriculum designs. Future research may address this challenge through multi-institutional collaborations, which could allow for larger samples without compromising internal validity. Another limitation of the present study is that it examined only L2-L1 CI in student interpreters. The interaction between participant profile (unbalanced bilinguals) and task features (L2-L1 CI) may have highlighted the importance of inhibition in this process. Given that previous research has found student interpreters to exhibit greater fluency in L2-L1 than in L1-L2 interpreting (Y. Lin et al., 2018), it is likely that interpreting direction imposes varying demands on cognitive resources, potentially highlighting different roles for specific cognitive abilities. Additionally, the cognitive load of CI has been found to be at least as high as, if not higher than, that of SI (Lv & Liang, 2018). Therefore, further work is needed to determine whether training on the three core EF components would yield different results in L1–L2 interpreting and in the SI modality, in order to assess whether the hypothesized role of inhibition observed in the current study remains valid across these contexts. In addition, due to practical constraints, this study could not adopt a single- or double-blind design. Therefore, we cannot entirely rule out the possibility that the observed improvements, particularly in EF abilities, were influenced by differences in motivation levels or expectations between trained and untrained participants. Nevertheless, we sought to minimize such effects through careful experimental planning and pre-test instructions designed to ensure that all participants were fully motivated for the research. Importantly, these expectancy effects are unlikely to account for the selective transfer observed in interpreting performance. Overall, despite the current study’s limitations, the findings hold promise for introducing targeted EF training to student interpreters’ training.

## 5. Conclusions and Implications

This study extends prior research on core EF in interpreting by investigating the distinct contributions of its diverse components: updating, inhibition, and TS. Separate training interventions for each component demonstrated positive effects on the corresponding trained EF component and on student interpreters’ interpreting performance; among interventions, only inhibition training reached statistical significance compared to the Control group. The present study’s asset lies in its three-month follow-up assessment, revealing a durable impact only for inhibition training. These results reinforce the strong and complex relationship between EF and interpreting processes and offer new insights into the nature of interpreting training.

While these findings may be highly relevant to student interpreter training, they do not advocate for immediately implementing EF (inhibition) training in the classroom or for replacing instruction in interpreting strategies. Interpreting, after all, is an activity of interpersonal interaction and intercultural communication (B. Wang, 2019), in which EF resources interact dynamically with interpreters’ contextual knowledge, strategic competence, and communicative awareness (Zhang & Yu, 2018). Nevertheless, based on the findings of this study, if cognitive training is to be introduced for student interpreters at the initial stage, inhibition training may yield more substantial effects.

As the first study to train all three EF components within an interpreting research framework, the present findings raise far more questions than they answer. Replication studies, preferably with larger samples, are crucial for further exploring the impact of distinct EF components on student interpreters, particularly within a developmental context and across different interpreting directions and modalities.

## Figures and Tables

**Table 1:** Summarization of key studies and insights for the present study.

Core EF Component Investigated	Author (Year)	Research Focus	Major Findings	Key Insights from the Literature for the Present Study
**Foundational research**				
updating	Dong et al. (2018)	Investigating whether updating and WM relate to student interpreters’ CI performance.	1. Student interpreters’ updating ability (measured by a visuo-spatial 2-back task) in both pre-test and post-test significantly predicted CI performance. 2. The relationship between WM (measured by an L2 listening span task) and CI performance was weaker.	Updating is closely related to interpreting performance and demonstrated strong predictive power, as supported by both cross-sectional and longitudinal studies.
	Y. Liu and Dong (2020)	Investigating the predictive power of updating for CI performance.	Student interpreters’ updating efficacy significantly predicted their CI performance.	
	Song et al. (2023)	Examining the role of EFs in bidirectional CI (Chinese-Japanese).	Updating exhibited a significant impact on both Japanese-to-Chinese and Chinese-to-Japanese interpreting, indicating that higher updating ability was associated with better interpreting performance.	
	Timarová et al. (2014)	Investigating the correlation between executive functions and simultaneous interpreting performance.	1. Interpreter’s better performance in the 2-back task is associated with a higher score on interpretation of numbers. 2. Interpreters who updated their memory more efficiently tended to use less extensive vocabulary.	
inhibition	Song et al. (2023)	Examining the role of EFs in bidirectional CI (Chinese-Japanese).	Inhibition showed a significant effect on Japanese-to-Chinese interpreting performance.	Inhibition may be significantly related to interpreting performance, although existing empirical evidence remains limited and inconclusive.
	Timarová et al. (2014)	Investigating the correlation between executive functions and simultaneous interpreting performance.	Interpreters with better performance in the Flanker task were more accurate in their interpretation of lexical items.	
	Xu (2012)	Examining the relationship between inhibition and interpreting processing.	Inhibition did not predict overall interpreting performance for either student or professional interpreters.	
TS	M. Liu (2014)	Examining the relationship between TS ability and CI performance.	There is a positive correlation between student interpreters’ TS ability and CI performance.	The role of TS is inconclusive, with findings varying across studies.
	Timarová et al. (2014)	Investigating the correlation between executive functions and simultaneous interpreting performance.	Interpreters who are better able to switch attention keep shorter EVS.	
	Song et al. (2023)	Examining the role of EFs in bidirectional CI (Chinese-Japanese).	No significant effects of shifting were observed in bidirectional CI.	
**Cognitive training research**				
updating	Öztürk (2018)	Examining the effects of updating training on CI performance.	1. 14 sessions of updating training improved participants’ CI performance. 2. The benefits of the training were maintained three months later. Both findings were supported by fNIRS evidence.	Cognitive training, which has thus far focused primarily on updating and WM in the literature, has been shown to improve interpreting performance, with some evidence suggesting the effects can be durable.
WM capacity	Zhang and Yu (2018)	Applying WM capacity and coordination training in SI teaching.	The training affirmed a positive effect, particularly in the beginning stage of learning.	

**Table 2:** Participants’ background information.

	Updating Group (*n* = 17)	Inhibition Group (*n* = 16)	TS Group (*n* = 13)	Control Group (*n* = 18)	*p*
Age	18.94 (0.56)	19.19 (0.66)	19.31 (0.75)	19.33 (0.59)	0.274
Sex, F/M	16/1	14/2	12/1	17/1	/
L2 age of acquisition	9.53 (2.18)	9.56 (2.25)	9.23 (0.83)	9 (0)	0.741
L2 proficiency ^1^	69.65 (5.81)	67.38 (9.95)	65.69 (6.29)	68.94 (7.12)	0.489
Handedness, right/left	17/0	15/1	13/0	18/0	/
Color-blind or not	not	not	not	not	/
WM span ^2^	52.59 (4.96)	52.63 (6.41)	51.46 (7.68)	51.17 (5.86)	0.864

^1^ Participants’ L2 proficiency was measured by their Test for English Major Band Four (TEM 4) scores. Administered nationally once a year, TEM 4 is a criterion-reference English proficiency test in China. It is one of the predominant English tests in China and is reasonably reliable and valid. ^2^ WM span was assessed by a shortened version of reading span task (60 rather than 75 sentences) (Dong et al., 2013).

**Table 3:** Overview of CI materials for T1, T2, and T3.

Feature	CI Material for T1	CI Material for T2	CI Material for T3
Speech Number	381	387	378
Total Duration (incl. interpreting time)	9 min 13 s	9 min 4 s	8 min 55 s
Domain	China-US cooperation	Chinese dialect	Kunming-Liverpool exchange
Words per Minute	120	126	122
Mean Sentence Length (words)	20.42	17.68	20

**Table 4:** ANOVA results: mean task difficulty as a function of Session for each training group.

Training Group	Training Task	*F*	*p*	η_p_^2^
Updating	Adaptive letter running task	91.252	<0.001 **	0.851
	Adaptive animal running task	87.259	<0.001 **	0.845
Inhibition	Adaptive Stroop task	29.985	<0.001 **	0.667
	Adaptive Flanker task	29.985	<0.001 **	0.667
TS	Adaptive task-cueing switching task	29.815	<0.001 **	0.665

** *p* < 0.01, two-tailed.

**Table 5:** Descriptive statistics of EF abilities and CI scores at three timeslots.

Task and Measure	Time	Updating Group	Inhibition Group	TS Group	Control Group
		*M* (*SD*)	*M* (*SD*)	*M* (*SD*)	*M* (*SD*)
Updating 2-back RT (ms)	T1	814.14 (221.38)	775.41 (217.39)	764.50 (195.09)	762.96 (289.58)
	T2	627.44 (160.28)	597.08 (151.60)	466.33 (72.29)	794.98 (250.74)
	T3	625.11 (187.34)	552.97 (164.16)	440.78 (67.38)	694.10 (247.08)
Inhibition Stroop effect (ms)	T1	93.67 (58.45)	75.79 (62.78)	111.70 (65.80)	88.44 (59.58)
	T2	80.76 (42.17)	16.28 (17.37)	52.85 (49.22)	100.80 (53.72)
	T3	95.08 (54.80)	30.39 (15.78)	43.77 (23.31)	104.34 (46.21)
TS					
Switching cost (ms)	T1	104.55 (155.71)	160.26 (189.31)	123.77 (113.27)	126.31 (151.85)
	T2	114.73 (99.80)	156.45 (170.10)	56.66 (45.16)	128.38 (142.34)
	T3	115.14 (91.22)	143.53 (239.46)	64.80 (68.86)	94.66 (118.67)
Mixing cost (ms)	T1	338.71 (149.92)	330.98 (130.63)	330.77 (95.81)	350.46 (186.58)
	T2	276.26 (103.58)	215.34 (103.17)	164.73 (27.50)	299.37 (127.86)
	T3	227.30 (130.65)	211.14 (102.81)	132.36 (61.63)	294.35 (100.82)
CI Performance scores	T1	51.94 (15.59)	50.44 (12.77)	50.23 (7.17)	50.44 (12.80)
	T2	63.85 (15.99)	65.37 (13.19)	63.52 (7.65)	53.00 (12.60)
	T3	54.36 (14.12)	64.18 (11.95)	52.88 (7.17)	52.03 (13.20)

**Table 6:** Practical effects and effect sizes for the CI task.

Analysis Type	Group/Contrast	Time Contrast	Mean Change/Difference [95% CI]	Effect Size [95% CI]
Within group changes				
	Updating (*n* = 17)	Δ(T2-T1)	11.91 [8.42, 15.40]	1.67 [0.93, 2.49]
		Δ(T3-T1)	2.41 [0.23, 4.59]	0.54 [0.05, 1.03]
	Inhibition (*n* = 16)	Δ(T2-T1)	14.94 [11.33, 18.54]	2.09 [1.21, 2.96]
		Δ(T3-T1)	13.75 [10.03, 17.46]	1.87 [1.05, 2.67]
	TS (*n* = 13)	Δ(T2-T1)	13.29 [10.76, 15.83]	2.97 [1.68, 4.23]
		Δ(T3-T1)	1.13 [1.21, 4.10]	1.04 [0.37, 1.68]
	Control (*n* = 18)	Δ(T2-T1)	2.56 [2.04, 3.07]	2.35 [1.44, 3.24]
		Δ(T3-T1)	1.59 [−0.05, 3.22]	0.46 [−0.12, 0.92]
Between-group contrast				
	Updating—Control	T2	10.85 [−1.13, 22.83]	0.74 [0.06, 1.41]
		T3	2.33 [−8.92, 13.57]	0.17 [−0.48, 0.81]
	Inhibition—Control	T2	12.37 [0.20, 24.54]	0.94 [0.24, 1.63]
		T3	12.15 [0.73, 23.58]	0.94 [0.24, 1.63]
	TS—Control	T2	10.52 [−2.37, 23.41]	0.95 [0.20, 1.68]
		T3	0.85 [−11.25, 12.96]	0.08 [−0.62, 0.77]

Note. CI = Confidence Interval. Hedges’ g is reported for interpretability. Conventional interpretations: small (~0.2), medium (~0.5), large (~0.8).

## Data Availability

The datasets analyzed in the current study are not publicly available since they are concerned with individual participants, and we made it clear in the informed consent form that their confidentiality would be ensured. But the data are available from the corresponding author upon reasonable request.

## Notes

1 Although the two terms EF and cognitive control are sometimes used interchangeably (e.g., Diamond, 2013), they differ in that EF is generally regarded as an umbrella term encompassing a range of cognitive processes, whereas cognitive control refers to the processes that guide behaviors, analogous to working definitions of EF today (Posner & Snyder, 1975; see also Goldstein et al., 2014, for a detailed review of EF models and definitions). In this study, EF is defined as a set of higher-order cognitive processes, with updating, inhibition, and task switching widely conceptualized as its core components (e.g., Diamond, 2013; Miyake et al., 2000; Song et al., 2023), while cognitive control denotes the online implementation of these processes.

2 In ACM, Dong and Li (2020) proposed an enhancement account for language control in interpreting, claiming a more practical way of modulating the two languages is the “enhancement of what is needed at the moment, letting irrelevant information simply be ignored” (p. 720). However, enhancement and inhibition may represent two alternative perspectives on the same target-accessing nature in interference inhibition tasks, such as the Stroop and Flanker tasks used in this study. Considering the academic positioning of inhibition as a core EF component and for consistency, the term inhibition will be used uniformly here.

3 After each speech segment, the audio already programmed a blank segment for interpreting, followed by the next segment of speech. Timeout interpreting for various reasons, such as a late start and hesitation, would be impossible as the next speech segment would already be playing by then. On average, the time allowed for interpreting is 1.5 longer than the speech segment duration.

4 Notes could relieve some of the memory pressure, but taking notes could be somewhat counterproductive, as retrieving appropriate symbols and abbreviations from one’s memory could take up too much processing capacity (Gile, 2009).

## Supplementary Materials

The following supporting information can be downloaded at https://www.mdpi.com/article/10.3390/bs15111477/s1: Description of EF ability assessment tasks; Full transcripts of the CI speeches; Detailed ANOVA result tables for all EF ability tasks.

## Abbreviations

The following abbreviations are used in this manuscript:

EF	Executive function
TS	Task switching
CI	Consecutive interpreting
SI	Simultaneous interpreting
WM	Working memory
ACM	The Attentional Control Model
EVS	Ear-voice span
ms	millisecond
PT	Presentation time
