# Supplementary material for: Effects of Core Executive Function Training on Student Interpreters’ Consecutive Interpreting
Source: Behav Sci (Basel). 2025 Oct 30;15(11):1477. doi: 10.3390/bs15111477 (PMC12649183; doi:10.3390/bs15111477)
Supplement: Supplementary file 1 [file behavsci-15-01477-s001.zip › behavsci-3876995-supplementary.pdf]

Supplementary Materials

Description of EF ability assessment tasks

Working memory updating: 2-back task

We measured working memory updating ability with different stimuli than those in the Updating training task. A sequence of numbers (1-9) presented successively for 500 ms, followed by a blank screen (2000 ms). Participants were required to press F if the current number was the same as the number presented two trials back in the sequence, starting from the third number. Press J in the other case. The test had two blocks, each 44 trials, of which the first two were non-response trials and 42 valid trials (a total of 84 trials will be analyzed). Across the task, 50% of the trials were match trials. The dependent measure was the average reaction time (RT) of the correct trials. A short RT reflects strong updating ability.

Inhibition: Stroop task

The inhibition ability was measured by the Stroop task. It adopted a similar structure to the adaptive Stroop training task but added a neutral condition, which consisted of the symbol "###" instead of the Chinese character. After a fixation point (500 ms), a stimulus appeared maximally for 1500 ms or until the participant made a response, whichever came first. Participants were required to report the color of the stimulus by pressing D for red, F for green, J for yellow, and F for blue. The test included three 36-trial blocks. Twelve trials of each condition (congruent, incongruent, and neutral) appeared randomly in each block, resulting in 108 trials across the task. The dependent variable was the Stroop effect, computed by the difference in RT between incongruent and congruent trials. A high score reflects weak inhibition ability.

Task switching: Digit-letter switching task

A digit-letter switching task was used to assess TS ability. It shared the same digit-letter combination stimulus as the TS training task but applied two different subtasks depending on the location of the stimulus. Each trial commenced with a fixation point "+" (1400 ms). Then, the target stimulus would randomly appear in the upper or lower half of a 2×2 square grid until the participant responded. Participants needed to perform the number magnitude task when the stimulus appeared in the upper part, deciding whether the value was smaller (1, 2, 3, 4) or larger (6, 7, 8, 9) than 5. The participants needed to press S for a number smaller than 5; otherwise, press A. When the stimulus appeared in the lower half, participants conducted a letter case task, in which they needed to judge whether the letter was lowercase (f, t, d, j) or uppercase (F, T, D, J) by pressing L key for lowercase letters and K for uppercases. The test included 20 blocks: 10 single-task blocks (8 trials each) and 10 mixed-task blocks (17 trials each). In the mixed block, the first trial was excluded from analysis, so the remaining 16 trials would form a pseudorandom combination of Task A and Task B, each presenting 8 times. The dependent variables were mixing cost— the average RT difference between non-switch trails in the mixed-task block and the single-task trials, and switching cost— the average RT difference between switch trials and non-switch trials in the mixed-task block. Higher mixing and switching costs indicate poorer TS ability.

Full transcripts of the consecutive interpreting speeches

Pre-test (T1)

More Americans are studying in China than in any other foreign country. Our goal is to send 100,000 students to China over the next four years. And I'm pleased to announce that the American private sector is showing increased support for the 100,000 Strong Initiative, with new commitments from several major companies.

And thank you so much, for your announcement of an additional 10,000 scholarships for Americans who wish to study in China. This is in addition to the 10,000 scholarships the Chinese Government offered last year, and we are very touched by this strong signal of support.

We also celebrate last month's announcement that New York University will open a campus in Shanghai in 2013. And we have with us today, the president of NYU. It is his vision to expand his university internationally while maintaining its reputation for excellence and academic freedom, and we're very excited about this endeavor which means so much to both of us.

I'm also pleased that we have a very robust set of exchanges--universities and dance companies, environmentalists and technology experts, Ping-Pong players, video game developers--all these and many more have a role to play in creating more understanding between our two countries. As you said, we all believe deeply that helping people expand their personal, commercial and professional exchanges really does pay enormous benefits.

We will not always agree. There is no doubt of that. I know of no two people -- not even within a family--that totally agree. But we believe strongly that working for greater understanding, finding new ways not only of communicating, but of cooperating, is in the best interest of our nations and our people. We think that the more our people learn to cooperate and collaborate, the more China and the

United States will be able to find solutions to many global challenges.

We both face climate changes issues, food security, environmental problems, educational issues, health care challenges, and we want to work to make sure we know the best solutions that can be applied everywhere. So, I thank you very much. You have understood this from the very beginning, and you have championed it within your government. I am pleased that both of us can continue this work together and I am excited by what lies ahead.

Post-test (T2)

Thank you for giving me a chance to explain our project. Basically, it is a website that details China's dialects. It aims to document China's many dialects via the use of recordings. In other words, we record native speakers in each of China's surviving dialect. We hope that in doing so, we can map many Chinese dialects and preserve this cultural heritage.

Why do we do this project? It all came about naturally. China has a huge variety of different dialects, but due to the common use of standard Mandarin in schools and general public places, these dialects are becoming increasingly difficult to preserve, and some dialects are even on the verge of becoming extinct. In Shanghai for example, many of the younger generation of locals are unable to speak the Shanghai dialect as well as their parents or grand-parents. This phenomenon is occurring, to a different extent, throughout China.

Keller and I are linguistic lovers. We are both interested in China's various local dialects, so we started thinking: Why don’t we ask people in different places to record stories in their own dialects and post them on a website so that we can provide a comprehensive, accessible database for preserving these dialects? We believe that preserving a language means preserving a history as languages are the carriers of history and culture.

Basically, we try to document the way Chinese people actually speak at home. We analyze the recordings of their speaking, eventually building up a database of linguistic features that will be able to be displayed dynamically. We're probably a year away from having this fully set up and usable. The core of the system is in place, but we only have one developer working on the whole system.

The project has received favorable responses right after it got started: many people submitted their recordings to our website, but unfortunately without transcriptions. That's quite frustrating: without text, some dialects are just like foreign languages and are totally incomprehensible. We use our blog to find volunteers. The volunteers help us to write down the recordings and find interviewees. Now we encourage contributors to upload their recordings and translate and edit the transcripts themselves. Most of the recordings on the website feature speakers telling a story in their various dialects, and last for around five minutes.

Delayed post-test (T3)

I am delighted to witness the signing of Liverpool's friendship agreement with China's Kunming City. The partnership will see the two cities build on existing ties and work together to create a positive, mutually beneficial relationship. The agreement is the result of a meeting between the vice mayor of the Kunming municipal government and the Liverpool mayor in September 2015. The meeting enabled the two sides to explore how the two cities can mutually benefit from closer ties.

Following on the positive discussions between the two city officials, we're delighted to be in Kunming to inaugurate our close partnership with this great city. I see our relationship with a city from China as a key element in Liverpool's future growth. This relationship creates a very positive platform for our two cities. It helps us to deliver sustainable economic and social benefits by collaborating on a broad range of activities, including trade and investment, science and technology, creative culture, and

tourism.

We’re excited at the prospect of welcoming the Kunming delegation to Liverpool for the International Festival for Business next year and look forward to further developing our relationship over the coming years. Liverpool is a vibrant and exciting city, which offers great opportunities for investment and we’re very pleased to be exploring this potential with Chinese organizations and businesses. Our visit to Kunming will help us identify great opportunities and strengthen our engagement with China.

In addition, there is fantastic potential to share and explore each other’s diverse cultural heritage, creating a rich, vibrant dialogue and positive exchange of ideas. And the two sides will collaborate on promoting the “Overseas Kunming Week” in Liverpool later this year. There is also the potential to host a Liverpool event in Kunming. The successful outcome of this visit boosts our cooperation and strengthens our cultural and commercial links with cities across southwest China.

The British Consulate has been working closely with Liverpool to support its growing city-to-city relationships in China through a year-long project that focused on urban development, healthcare, and creative culture. The two cities have already worked together on environmental protection, medical insurance, and key technologies. Liverpool Wild Flower Protection Center also signed a memorandum of understanding with Kunming. A medical delegation from Liverpool visited Kunming in March 2015.

Table S1. Practical effects and effect sizes for the 2-back task.

Analysis type	Group/Contrast	Time contrast	Mean change/difference [95% CI]	Effect size [95% CI]
Within group changes				
	Updating (n = 17)	Δ (T2-T1)	-186.70 [-248.38, -125.03]	-1.48 [-2.15, -0.79]
		Δ (T3-T1)	-189.03 [-288.46, -89.59]	-0.93 [-1.48, -0.37]
	Control (n = 18)	Δ (T2-T1)	32.02 [-47.50, 111,54]	0.19 [-0.26, 0.63]
		Δ (T3-T1)	-68.86 [-181.28, 43.55]	-0.29 [-0.74, -0.16]
Between-group contrast				
	Updating - Control	T2	-167.54 [-331.21, -3.89]	-0.77 [-1.44, -0.09]
		T3	-68.99 [-239.90, 101.02]	-0.31 [-0.96, 0.35]

Note. CI = Confidence Interval. Hedges' g is reported for interpretability. Conventional interpretations: small (~0.2), medium (~0.5), large (~0.8).

Table S2. Practical effects and effect sizes for the Stroop task.

Analysis type	Group/Contrast	Time contrast	Mean change/difference [95% CI]	Effect size [95% CI]
Within group changes				
	Inhibition (n = 16)	Δ (T2-T1)	-59.51 [-96.28, -22.73]	-0.82 [-1.36, -0.26]
		Δ (T3-T1)	-45.39 [-74.56, -16.23]	-0.79 [-1.32, -0.24]
	Control (n = 18)	Δ (T2-T1)	12.36 [-12.67, 37.38]	0.24 [0.22, 0.68]
		Δ (T3-T1)	15.90 [-11.00, 42.79]	0.28 [-0.17, 0.73]
Between-group contrast				
	Inhibition - Control	T2	-84.52 [-124.86, -44.18]	-2.02 [-2.83, -1.19]
		T3	-73.94 [-111.17, -36,71]	-2.04 [-2.86, -1.21]

Table S3. Practical effects and effect sizes for mixing cost of TS task.

Analysis type	Group/Contrast	Time contrast	Mean change/difference [95% CI]	Effect size [95% CI]

Within group changes				
	TS (n = 13)	Δ (T2-T1)	-166.04 [-227.46, -104.62]	-1.53 [-2.31, -0.73]
		Δ (T3-T1)	-198.41 [-267.32, -129.50]	-1.63 [-2.44, -0.80]
	Control (n = 18)	Δ (T2-T1)	-51.09 [-102.36, 0.17]	-0.47 [-0.94, 0.00]
		Δ (T3-T1)	-56.12 [-134.66, 22.43]	-0.34 [-0.79, 0.12]
Between-group contrast				
	TS - Control	T2	-134.64 [-235.46, -33.82]	-1.32 [-2.08, 0.54]
		T3	-161.99 [-265.36, -58.61]	-1.82 [-2.64, -0.97]

Table S4. Practical effects and effect sizes for switching cost of TS task.

Analysis type	Group/Contrast	Time contrast	Mean change/difference [95% CI]	Effect size [95% CI]
Within group changes				
	TS (n = 13)	Δ (T2-T1)	-67.11 [-133.38, -0.84]	-0.57 [-1.12, -0.01]
		Δ (T3-T1)	-58.96 [-121.56, 3.63]	-0.53 [-1.07, 0.03]
	Control (n = 18)	Δ (T2-T1)	2.06 [-77.09, 81.22]	0.01 [-0.43, 0.45]
		Δ (T3-T1)	-31.65 [-117.34, 54.04]	-0.18 [-0.62, 0.27]
Between-group contrast				
	TS - Control	T2	-71.71 [-197.49, 54.06]	-0.62 [-1.33, 0.10]
		T3	-29.86 [-175.46, 115.74]	-0.29 [-0.98, 0.41]
